# General practitioners’ decision-making strategies in the pharmacological treatment of musculoskeletal pain: A qualitative interview study

**DOI:** 10.1080/13814788.2025.2536764

**Published:** 2025-08-01

**Authors:** Nele Kornder, Victoria Jessica Hill, Sophia Naomi Groffebert, Annette Becker, Annika Viniol, Nicole Lindner

**Affiliations:** Department of Primary Care, Philipps-Universität Marburg, Marburg, Germany

**Keywords:** Musculoskeletal pain, General practitioners, analgesic prescribing, shared decision-making

## Abstract

**Background:**

Musculoskeletal pain is a leading reason for primary care visits and often requires pharmacological treatment. Despite rising prescription rates for non-opioid analgesics in Germany, little is known about GPs’ broader prescribing behaviour beyond opioid-related discussions. Understanding how GPs navigate pain management is key to supporting evidence-based prescribing.

**Objectives:**

This study explored GPs’ decision-making strategies when prescribing for musculoskeletal pain and identified clinical challenges.

**Methods:**

A qualitative study using semi-structured interviews was conducted with 15 GPs from Central and Northern Hesse, Germany. Participants were purposively recruited via a regional practice network. Interviews were analysed using Braun and Clarke’s thematic analysis, applying a combined deductive–inductive approach.

**Results:**

Five major themes emerged: (1) prescribing approaches, (2) medication preferences, (3) doctor–patient relationship, (4) addressing psychosomatic factors, and (5) support needs. GPs preferred cautious prescribing, favouring metamizole and NSAIDs over opioids. Chronic pain was viewed as complex and required individualised, multimodal treatment and shared decision-making. Decision-making strategies were mainly shaped by guidelines like the WHO analgesic ladder and personal clinical experience; other guidelines were rarely mentioned. The doctor–patient relationship was considered essential, particularly in chronic pain contexts. Challenges included managing psychosomatic aspects and aligning treatment expectations.

**Conclusion:**

GPs’ prescribing decisions are shaped by a combination of clinical judgement, patient dynamics, and systemic factors. The findings highlight the need for practical support tools that are integrated into daily workflows and emphasise shared decision-making, especially for chronic pain management. These insights can inform future interventions aimed at optimising prescribing practices in primary care.

## Introduction

Musculoskeletal pain is a leading cause of primary care consultations [1,2] and often necessitates pharmacological treatment. In 2021, ibuprofen was the most prescribed drug in Germany, with metamizole also ranking high despite concerns about its side effects (e.g. agranulocytosis) [3]. The long-term use of analgesics poses considerable challenges, particularly concerning their efficacy, safety, and potential for dependency [4,5]. However, analgesics are indispensable in the treatment of acute pain and in managing exacerbations of chronic pain, as they have the potential to provide pain relief and help improve patients’ quality of life [6].

While non-opioid analgesic prescriptions have increased [3,7], the latest data from 2025 show a decline in opioid prescriptions in Germany between 2005 and 2020 [8]. There is a considerable amount of research on the use of opioids in the treatment of chronic pain [9–11], yet prescribing practices remain controversial due to concerns about long-term use, dependency, and misuse. Qualitative studies offer important insights into this ongoing debate. These studies consistently highlight healthcare providers’ perceptions of significant barriers to prescribing opioids. They also emphasise the need for improved education and support systems to promote safe and effective prescribing practices [6,12].

Yet despite rising prescription rates for non-opioid analgesics, there is limited research on the broader prescribing behaviours of general practitioners (GPs) beyond. The lack of qualitative data leaves a significant gap in understanding the decision-making processes, challenges, and support needs of GPs regarding the spectrum of analgesic options. This gap is particularly pronounced in Germany, which has a distinct prescribing landscape compared to many English-speaking countries – most notably characterised by the widespread use of metamizole.

The aim of this study was to explore the decision-making strategies of GPs when prescribing medication for musculoskeletal pain and to identify the challenges they face. Understanding these barriers will help develop targeted support to optimise prescribing practices and improve patient care.

## Methods

### Study design

We conducted a semi-structured interview study with GPs to identify barriers and facilitators in the prescribing process. The findings will contribute to the creation of a decision-making aid to support evidence-based prescribing for musculoskeletal pain (e.g. an app or web-based platform).

To capture the complexity of GPs’ prescribing practices and reasoning, we chose a qualitative approach. Informed written consent was obtained from all participants following an explanation of the study’s aims and potential outcomes, in accordance with the principles of the Declaration of Helsinki [13]. The Ethic Board of the University of Marburg approved the study (ethics approval ID: 23–95). Members of a patient advisory board were involved in the study; for example, they provided feedback on the interview guide, suggested additional questions (e.g. on wait times, digital tools, pharmacy counselling), and advised on recruitment strategies. The research team consisted of two medical doctoral candidates (VH, SG), two academic GPs with experience in qualitative research (NK, NL), and two senior researcher who are also practicing GPs (AB, AV). All researchers engaged in reflexivity by being mindful on their personal experiences with pharmacological treatment of chronic musculoskeletal pain and, where relevant, their work as practicing GPs, to enhance awareness of potential biases during data collection and analysis. The study process underwent internal peer review within the research team and external review through a presentation at the German GP Conference.

### Study sample

The interview study included GPs practicing in Central and Northern Hesse, Germany. Participants were recruited through the regional research practice network, which comprises GP practices that can be contacted for participation in research studies conducted by our institute. Invitations were sent via telephone, email, or postal mail, along with study materials including a consent form and a sociodemographic questionnaire. Inclusion criteria required GPs to have been practicing for at least one year. Trainees were excluded. Efforts were made to ensure diversity among participants regarding practice settings (single/group practices), geographic locations (urban/rural areas), and gender balance.

Recruitment was concluded once sufficient data had been collected to answer the study question, and no new themes emerged.

### Data collection

The interview guide was developed based on a comprehensive review [e.g. 4–6,9,12,14] of the existing literature on prescribing practices for musculoskeletal pain, with a focus on non-opioid analgesics. The questions aimed to explore GPs’ reasoning, decision-making processes, perceived challenges, and contextual influences on prescribing behaviour. These topics were chosen to better understand the complexity of everyday prescribing in primary care.

Analgesics were defined as pharmacological agents used to relieve musculoskeletal pain, including non-opioid and opioid medications (e.g. non-steroidal anti-inflammatory drugs (NSAIDs), metamizole, and opioids), as well as co-analgesics (e.g. antidepressants, anticonvulsants). The interview guide was refined based on feedback from the patient advisory board and from initial interviews to ensure clarity and depth of data collection. Sociodemographic data were collected using a questionnaire.

Participants were recruited between June 2023 and November 2024. Interviews were conducted between July 2023 and November 2024 by VH and SG at locations chosen by the participants, such as their practice, telephone or video conference. All interviews were conducted in German, as all participants were fluent in the language. An overview of main topics and corresponding sample questions are presented in Box 1.

Box 1. Interview guide for GPs. Overview of main topics and corresponding sample questions discussed in the interviews.

**Table:** 

**Main topic:** Approach to acute musculoskeletal pain **Sample question** ‘A young man (30 years old) complains about pain in the upper back/shoulder-neck region. He rates the intensity on a numerical rating scale as 5. The pain has been present for 7 days and shows no improvement. How would you proceed?’
**Main topic:** Approach to chronic musculoskeletal pain **Sample question** ‘Assuming the young man is an elderly woman (75 years old) with various pre-existing conditions, who also presents with back pain. However, she has been suffering from the pain for several months. How would you proceed with this patient?’
**Main topic: **Patient characteristics/factors influencing prescription of pain medication **Sample question** ‘Which patient characteristics/ factors are crucial for you when prescribing pain medication?’
**Main topic:** Psychosomatic aspects regarding the treatment of musculoskeletal pain **Sample question** ‘To what extent do you consider psychosomatic aspects when prescribing pain medication?’
**Main topic: **Shared decision-making vs. paternalistic therapy decisions **Sample question** ‘To what extent do you decide on treatment decisions together with patients?’
**Main topic: **Role of GP-patient relationship when prescribing pain medication **Sample question** ‘What role does the GP-patient relationship take when prescribing?’
**Main topic**: Personal strategies when prescribing pain medication **Sample question** ‘What are your personal strategies that help you to prescribe pain medication? What literature/reference works etc. do you use?’
**Main topic:** Personal decision-making strategies for prescribing common pain medications such as NSAIDs, paracetamol, metamizole and opioids **Sample question** ‘What kind of patients do you prescribe NSAID to?’ ‘How do you choose the dosage?’ ‘To what extent do you check after a certain time whether the patient is still dependent on the medication?’
**Main topic: **Personal attitude towards the use of co-analgesics for musculoskeletal pain **Sample question** ‘Do you use co-analgesics in addition to traditional painkillers for the treatment of musculoskeletal pain? Which ones?’
**Main topic: **Concept for inadequate pain control **Sample question** ‘How do you deal with inadequate pain control? What is your personal concept for escalation?’
**Main topic: **Uncertainties and errors associated with pain medication for musculoskeletal pain **Sample question** ‘Are there situations in which you feel or have felt unsure when prescribing pain medication? You are also welcome to tell us about such a situation.’
**Main topic: **The need for and form of support in the context of prescribing painkillers for musculoskeletal pain **Sample question** ‘Is there anything that would make prescribing painkillers easier for you? What ideas do you have for such a program?’

### Data analysis

Interviews were audio-recorded and transcribed verbatim. Quotes are referenced by pseudonyms (e.g. ‘GP_1’), with additional demographic information (e.g. age, gender) provided separately in a participant characteristics table (Table 1). Quotes were translated into English with the assistance of artificial intelligence (OpenAI, ChatGPT-4) to ensure accuracy and consistency in the translation process. Data were managed using MAXQDA (2022). The analysis followed Braun and Clarke’s six-step thematic analysis methodology [15,16], employing a deductive–inductive approach. While the interview guide informed initial theme development (deductive), participant responses allowed for new themes to emerge (inductive).

**Table 1.** Characteristics of interview partners.

GPs characteristics and demographics (*n* = 15)				
GP^a^	Age (years)	Time working as GP (years)	Setting	Practice form
GP_1	61	ï¹¥15 years	Rural	Individual practice
GP_2	49	6–15 years	Urban	Individual practice
GP_3	48	﹤5 years	Rural	Group practice
GP_4	70	ï¹¥15 years	Rural	Group practice
GP_5	62	6–15 years	Urban	Individual practice
GP_6	56	ï¹¥15 years	Urban	Group practice
GP_7	38	6–15 years	Rural	Group practice
GP_8	45	6–15 years	Rural	Group practice
GP_9	62	ï¹¥15 years	Rural	Group practice
GP_10	56	ï¹¥15 years	Urban	Group practice
GP_11	55	ï¹¥15 years	Rural	Group practice
GP_12	57	ï¹¥15 years	Urban	Group practice
GP_13	40	6–15 years	Rural	Group practice
GP_14	64	ï¹¥15 years	Urban	Group practice
GP_15	56	ï¹¥15 years	Rural	Group practice

First, VH and SG familiarised themselves with the data by listening to audio recordings, reading transcripts, and taking analytical notes. Relevant text passages were identified, and initial codes were assigned. These codes were grouped into overarching themes that were distinct and substantiated by underlying codes. The coding framework was refined iteratively through discussions within the research team (NK, NL). Emerging themes were visualised using thematic maps, which were iteratively optimised to capture the relationships between themes and subthemes. Sociodemographic data were analysed descriptively.

## Results

A total of 15 GPs (6 women and 9 men) were interviewed. The interviews were conducted via telephone [9], in person [3], or via video conference [3]. Interviews lasted between 17 and 55 min. GP ages ranged from 30 years to over 60 years. Characteristics of the GPs are summarised in Table 1.

Patterns in the data were analysed and resulted in the following themes:

1. Prescribing Approaches in Pain Management.
2. Medication Preferences.
3. The Role of the Doctor–Patient Relationship.
4. Addressing Psychosomatic Components.
5. Support Need in Pain Medication.

**Figure 1.**
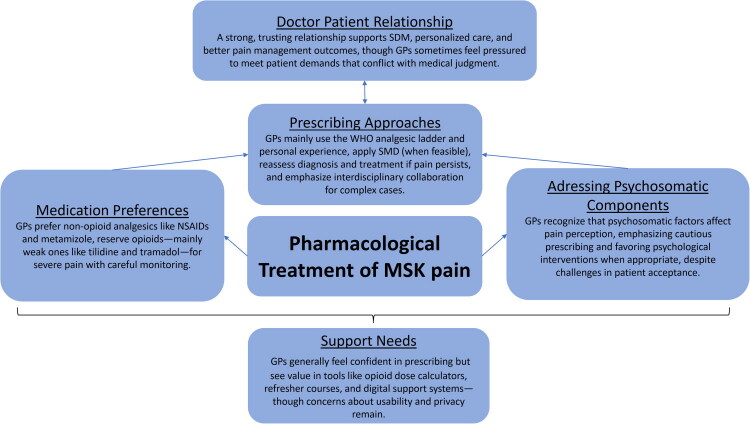
Overview of factors influencing pharmacological treatment of musculoskeletal pain. Interview partners expressed: Prescribing approaches; medication preferences; doctor–patient relationship; addressing psychosomatic components; support needs. SDM: Shared-Decision-Making.

Figure 1 provides an overview of the important factors influencing pharmacological treatment of musculoskeletal pain.

### Prescribing approaches in pain management

GPs most commonly referred to the World Health Organisation (WHO) analgesic ladder as a guiding framework, complemented by personal experience when making prescribing decisions. Long-standing habits and knowledge from years of practical work played a central role, with many relying on a familiar set of medications they had seen work reliably in the past. Formal resources like guidelines issued by the German College of General Practitioners and Family Physicians (DEGAM) or databases were rarely mentioned, typically consulted only in complex cases or when deviating from standard routines. Their approach varied depending on whether the pain was acute or chronic.

Efforts were made to engage patients in shared decision-making (SDM), ensuring they are actively involved in treatment choices:

What helped you last time? Uh, yes, and if that’s something reasonable, then of course I take that into account and advise them to do the same again. So, this almost always happens in consultation with the patient (…). (GP_1)

However, their approaches varied: While some reported prioritising patient preferences as long as they posed no harm, others stated that they maintained greater control over prescribing decisions, especially when they perceived SDM as too time-consuming or impractical, such as in cases of severe anxiety or dementia:

[…] that is my decision. Sometimes, that [SDM] would take too long. (GP_14)

[…] with anxious patients, you sometimes cannot do that [involve them in the decision-making process] because they are completely unsettled. (GP_12)

In cases when pain treatment proves insufficient, GPs stated they first reassessed the diagnosis and to adjust the treatment plan (medication type/dosage, introducing co-analgesics and non-pharmacological interventions):

Of course, one must check: Is the diagnosis correct? Do the resources of the practice suffice to meet the patient’s needs? Does the patient need to be referred to a specialist? Is imaging necessary? That’s clear, right? So first, verify the diagnosis. (GP_3)

Furthermore, GPs emphasised the importance of interdisciplinary collaboration, particularly in cases of persistent or complex pain. Multimodal pain therapy – combining pharmacological and non-pharmacological approaches – was seen as a valuable option when treatment within primary care reached its limits. While some GPs actively referred patients to pain specialists or inpatient programs, others expressed reservations, for example due to concerns about overprescription of opioids.

### Medication preferences

GPs tailored their medication choices based on clinical needs, balancing efficacy and safety. In general, the interviewed GPs appeared to have clear internalised dosing schemes for commonly used analgesics.

Non-opioid analgesics such as NSAIDs, metamizole, and paracetamol were generally preferred, with opioids reserved for severe pain and used sparingly. NSAIDs were the most commonly prescribed for acute and exacerbated chronic pain, especially in younger, healthier patients, with careful use in older or comorbid patients due to risks like gastrointestinal or cardiovascular side effects. Ibuprofen and diclofenac were the preferred options, chosen partly based on patient experience and tolerability:

My preferred medications are Ibuprofen and Diclofenac, which I often use interchangeably, as some patients tolerate Diclofenac better, while others do better with Ibuprofen. […] I also rely on the patients’ own experiences. (GP_6)

Metamizole was regarded as an effective and well-tolerated option, mainly for severe pain and short-term use, with patient education about rare but serious side effects like agranulocytosis.

Paracetamol was generally not a first choice for musculoskeletal pain but used in cases of contraindications to NSAIDs or metamizole, or for mild pain and fever.

Opioids seemed to be reserved for severe pain and used very rarely, with a strong focus on monitoring to prevent misuse:

Super, super, super, super rare. I would say it [prescription of opioids] practically never happens. Well, maybe in cases of persistent severe pain despite a combination of NSAIDs and metamizole. (GP_7)

When opioids were prescribed, GPs generally preferred weak opioids, typically as an add-on to NSAIDs or metamizole. Tilidine and tramadol were most commonly mentioned, valued for their tolerability and, in the case of tilidine, the reduced risk of misuse due to the inclusion of naloxone. Strong opioids were used more cautiously and primarily in specific cases, such as end-of-life care or when other treatments failed. GPs emphasised the importance of patient education, particularly regarding side effects (e.g. nausea and driving impairment). They seemed to be very cautious about long-term use due to the risk of dependence and stated to avoid their prescribing for extended periods unless absolutely necessary. Overall, opioids seemed to be prescribed far less frequently than NSAIDs or metamizole, with the GPs being highly selective and controlled in their use. Strong opioids were reserved for exceptional cases (e.g. end-of-life care).

Co-analgesics (antidepressants, anticonvulsants) were used selectively, mainly for chronic pain with ‘*psychosomatic components*’ (GP_6). GPs emphasised the need for monitoring (e.g. electrocardiogram [EKG]) and noted challenges including side effects, patient reluctance, and concerns about stigma:

Many pain patients are also not willing to take psychiatric medication, because they feel that it means they are not taken seriously. (GP_4)

Non-drug therapies, such as physiotherapy and complementary medicine, also seemed to play a major role, particularly when aligned with patient preferences.

### The role of the doctor–patient relationship

A strong doctor–patient relationship was seen as key to effective pain management, especially in chronic pain. Trust and support were described as central for SDM, adherence, and outcomes:

That [doctor–patient relationship] is incredibly important. I have been in practice for 20 years now, and the trust that patients place in me is enormous […]. (GP_1)

GPs stated that in primary care, knowing patients over time helps doctors better understand their needs and medical histories, making treatment more personalised. Long-term relationships also allow GPs to consider psychosocial factors that influence pain management:

[the doctor–patient relationship plays] a big role. A very big role. I think this is also our strength as GPs—that we don’t just look at the pain or the physical symptoms, but we also know if the patient’s husband has been unfaithful or if their daughter is struggling with drug addiction. These things always play a role in how we perceive and approach the patient. Or take their job, for example – how demanding is it? […] These are all factors that influence treatment decisions, sick leave duration, and pain management! (GP_15)

GPs aimed to work as partners with patients, balancing guidelines with individual preferences. However, some reported feeling pressured to fulfil treatment requests that conflicted with medical judgement:

Yes, I think it becomes challenging when you’re confronted with expectations that you’re reluctant to fulfill, but you still do so anyway – that’s the difficult part. (GP_7)

Often it seemed to shift towards patients dictating their desired treatments, positioning doctors almost in a service-provider role, which can create challenges in balancing medical expertise with patient expectations.

I have no objections at all – they should get and take whatever relieves their pain. (GP_5)

The framing of the GP as a service-provider was described by some participants and may be relevant to deprescribing efforts – particularly for medications with dependency potential – although this aspect was rarely addressed in the interviews.

### Addressing psychosomatic components

GPs highlight the interplay between pain and psychosomatic factors, stressing the need for careful diagnosis and tailored interventions. They were aware that patients may experience altered pain perception due to psychological factors, and that medications like painkillers can only offer limited relief in such cases.

Where one tends to be more cautious, because you know that this might be someone who, due to their psychological situation, might have a completely different perception of pain, and you also notice that the pain medication doesn’t work in the typical way. (GP_13)

In managing these aspects GPs stressed the need of a multidisciplinary approach that can significantly impact treatment outcomes. For patients with psychosomatic components, GPs mentioned to prioritise psychological interventions over medications:

Yes, if I have the impression that it’s more in the depressive direction, then I am more cautious with pain medication. (GP_11)

Treating patients with musculoskeletal pain with a psychosomatic component was described as challenging. Patients may not feel taken seriously when this aspect is raised, and GPs may struggle to fully acknowledge the psychosomatic factors.

### Support needs in pain medication therapy

Some GPs saw no need for additional assistance, others welcomed measures to streamline prescribing processes. Monitoring of opioid prescriptions, as well as tools for dosage conversion, was seen as potentially useful, particularly given the risk of dosing errors that can occur when adjusting pain medication regimens:

Conversion tables are available on the internet, but they are relatively difficult to find and complicated to calculate. If there were some kind of calculator for this… (GP_4)

Despite these potential benefits, all of the interviewed GPs seemed to have clear frameworks and established practices in mind for choosing pain medications and their dosages, suggesting that they generally feel confident in their decision-making. They appeared to need less advice on when and how to prescribe specific medications, and were often guided by their clinical experience rather than additional support.

Some physicians emphasised the need for refresher courses, e.g. in physical examination techniques to evaluate the pain. Opinions were divided on a digital decision-support system, with concerns about data privacy and increased workload. Any potential support program would need to account for individual patient factors and red flags to ensure safe and effective pain management:

Basically, it’s certainly not a bad idea to have something where you can enter symptoms and somehow rule out red flags or similar issues. (GP_8)

They emphasised the need for tools that provide updated knowledge, facilitate SDM, and integrate seamlessly into clinical workflows. However, scepticism remains regarding the practicality and efficacy of such interventions.

## Discussion

### Summary

Our study provides a comprehensive overview of the decision-making approaches regarding the management of musculoskeletal pain in primary care. The GPs involved in our study had clear frameworks and preferences when selecting pain medications. NSAIDs were generally the first choice for managing musculoskeletal pain. Metamizole was considered a secondary option, typically used when NSAIDs were ineffective or contraindicated. Opioids were rarely prescribed and only in very specific cases, reflecting the GPs’ cautious approach to these medications. When treatment was insufficient, GPs reassessed diagnoses, adjusted plans, and considered specialist referrals or multimodal therapy. SDM was key but challenging, especially when patient expectations conflicted with best practices. While opinions on support tools varied, some saw potential benefits in digital aids for prescribing, though scepticism about their practicality remained.

### Comparison with existing literature

The interviewed GPs stressed the necessity of an individualised approach, with many medications mentioned as part of their pain management strategies. Clear ideas were expressed about when and which medication to prescribe, reflecting their well-established routines and preferences. Previous studies exploring prescription behaviour in primary care also indicate that while guidelines provide a framework, real-world prescribing decisions are shaped by clinical experience, patient preferences, and contextual factors [14,17]. For instance, some GPs in our study viewed tramadol as a well-tolerated option, which contrasts with more cautious perspectives due to its side effect profile [18]. This highlights how local practice norms and outpatient contexts may influence medication preferences and perceptions of tolerability.

Surprisingly, many GPs in our study reported using the WHO analgesic ladder as their primary framework for pharmacological pain treatment, while references to more specific clinical guideline were rare. This is noteworthy given that the WHO ladder was originally developed for cancer pain management and does not specifically address the nuances of musculoskeletal pain. In contrast, guidelines like the German National Care Guideline (NVL) Low Back Pain [19] or the National Institute for Health and Care Excellence (NICE) guideline [20] are tailored to incorporate biopsychosocial dimensions and non-pharmacological, but were hardly mentioned strategies. The limited use of such targeted guidelines suggests a potential gap between available resources and routine practice. This highlights the need to strengthen awareness and implementation of condition-specific guidelines in general practice to improve patient care. Furthermore, GPs highlighted the importance of actively engaging patients in treatment decisions through a SDM approach to ensure that medical best practices align with individual needs and expectations. This finding is consistent with existing research on patient-centered communication in primary care, which underscores the significance of empathy and psychosocial considerations in optimising treatment outcomes [21]. A trusting doctor–patient relationship is fundamental to this process and is regarded as a crucial component in managing chronic pain within primary care settings. However, some GPs reported that the doctor–patient relationship can become strained, particularly when patients have strong expectations regarding analgesic prescriptions. Patients often enter consultations with fixed expectations—whether for specific prescriptions or treatments—placing pressure on doctors to fulfil demands rather than guide care based on clinical judgement. This ‘service-provider dynamic’ may hinder deprescribing efforts, especially for medications with dependency potential such as opioids or gabapentinoids. Similar tensions have been described in an interview study on exercise counselling for patients with chronic back pain. Here, too, GPs reported that their role was at times confined to fulfilling patient expectations, which negatively impacted the doctor–patient relationship [22]. Similarly, a study on chronic low back pain patients and their physicians highlighted a mismatch between patients’ reliance on biomedical models and the physicians’ adherence to biopsychosocial approaches, contributing to communication barriers and treatment discordance [23].

Furthermore, our qualitative study revealed that GPs approach opioid prescriptions with great caution, favouring NSAIDs or alternatively metamizole whenever possible. Uncertainties in the prescribing of opioids and the associated need for support have also been highlighted in previous qualitative studies [6,12]. This cautious prescribing approach contrasts with prescribing patterns in countries like the United States (U.S.), where opioids have historically been more readily used for pain management [24,25]. One key factor influencing this difference might be the availability of metamizole in Germany, which serves as an alternative to NSAIDs and is often preferred due to its strong analgesic properties and lower gastrointestinal risk. This is in line with prior research demonstrating the continued high prescription rates of metamizole in general practice over the past decade [26]. The persistence of its use suggests that GPs perceive it as an effective option, even though the German Federal Institute for Drugs and Medical Devices (BfArM) has issued warning about rare but serious adverse effects like agranulocytosis. In many other countries, including the U.S., metamizole is either restricted or unavailable, potentially contributing to a greater reliance on opioids for pain relief. This underscores the impact of national pharmaceutical regulations on prescribing behaviours and highlights the importance of exploring diverse pain management options.

### Strength and limitations

A major strength of this study is that it explores GPs’ broader perspectives on prescribing pain medications for musculoskeletal pain in Germany – a topic that has received little qualitative attention to date. The sample included GPs of varying gender, age, and practice settings, which supports the transferability of the findings.

Semi-structured interviews allowed for an in-depth exploration of GPs’ experiences, capturing the complexity of the issue.

Despite these strengths several limitations have to be acknowledged: As with other qualitative studies, transferability to different contexts may be limited. Additionally, there is a potential for research bias during data collection and interpretation. However, this was mitigated through close supervision of the interviewers by senior researchers and ongoing reflexive team discussion to enhance trustworthiness. Another limitation is our focus on pharmacological treatment; aspects such as identifying the underlying cause of pain or using non-drug therapies may not have been fully captured, which may limit the comprehensiveness of our findings.

## Conclusion

This study highlights the complexity of pain management in primary care, where GPs balance clinical frameworks, patient expectations, and structural constraints. Shared decision-making plays a central but sometimes difficult role, particularly when expectations and best practices diverge. While GPs appeared to have clear internal frameworks regarding the choice and dosing of analgesics – and therefore expressed little need for support in this area – they emphasised greater challenges in aligning patient expectations with evidence-based recommendations. The frequent reference to the WHO analgesic ladder, while more specific guidelines were rarely mentioned, may point to the persistence of familiar but potentially outdated prescribing habits. GPs expressed mixed views on a potential supporting tool. To be effective, it must be user-friendly, time-efficient, and seamlessly fit into clinical workflows.

## Data Availability

The data supporting this study are not publicly available, but may be provided by the corresponding author upon reasonable request.
